# Nuclear HER4 mediates acquired resistance to trastuzumab and is associated with poor outcome in HER2 positive breast cancer

**DOI:** 10.18632/oncotarget.1904

**Published:** 2014-04-17

**Authors:** Siti Norasikin Mohd Nafi, Daniele Generali, Gabriela Kramer-Marek, Merel Gijsen, Carla Strina, Mariarosa Cappelletti, Daniele Andreis, Syed Haider, Ji-Liang Li, Esther Bridges, Jacek Capala, Roxanis Ioannis, Adrian L Harris, Anthony Kong

**Affiliations:** ^1^ Human Epidermal Growth Factor Group, Department of Oncology, Weatherall Institute of Molecular Medicine, University of Oxford, John Radcliffe Hospital, Oxford, UK; ^2^ Growth Factor Group, Department of Oncology, Weatherall Institute of Molecular Medicine, University of Oxford, John Radcliffe Hospital, Oxford, UK; ^3^ National Institutes of Health, Radiation Oncology Branch, Bethesda MD, US; ^4^ U.O. Multidisciplinare di Patologia Mammaria, U.S Terapia Molecolare e Farmacogenomica, A.O. Instituti Ospitalieri di Cremona, Viale Concordia 1, Cremona, Italy; ^5^ Department of Cellular Pathology, Oxford University Hospitals and Oxford Biomedical Research Centre, Oxford, UK; ^6^ Institute of Cancer Research, Division of Radiotherapy and Imaging, 15 Cotswold Road, Belmont, Sutton, Surrey, UK

**Keywords:** breast cancer, HER4, HER2, trastuzumab, resistance

## Abstract

The role of HER4 in breast cancer is controversial and its role in relation to trastuzumab resistance remains unclear. We showed that trastuzumab treatment and its acquired resistance induced HER4 upregulation, cleavage and nuclear translocation. However, knockdown of HER4 by specific siRNAs increased trastuzumab sensitivity and reversed its resistance in HER2 positive breast cancer cells. Preventing HER4 cleavage by a γ-secretase inhibitor and inhibiting HER4 tyrosine kinase activity by neratinib decreased trastuzumab-induced HER4 nuclear translocation and enhanced trastuzumab response. There was also increased nuclear HER4 staining in the tumours from BT474 xenograft mice and human patients treated with trastuzumab. Furthermore, nuclear HER4 predicted poor clinical response to trastuzumab monotherapy in patients undergoing a window study and was shown to be an independent poor prognostic factor in HER2 positive breast cancer. Our data suggest that HER4 plays a key role in relation to trastuzumab resistance in HER2 positive breast cancer. Therefore, our study provides novel findings that HER4 activation, cleavage and nuclear translocation influence trastuzumab sensitivity and resistance in HER2 positive breast cancer. Nuclear HER4 could be a potential prognostic and predictive biomarker and understanding the role of HER4 may provide strategies to overcome trastuzumab resistance in HER2 positive breast cancer.

## INTRODUCTION

HER4 is a cell surface receptor that belongs to the human epidermal growth factor receptor (HER/ErbB) family [1, 2]. The signaling by HER4 is initiated through ligand binding of heparin-binding EGF, betacellulin and epiregulin (co-ligands with EGFR/HER1) [3, 4] or heregulin (NRG1-4) [3, 5, 6]. HER4 activation by its different ligands results in variable combination of HER4 as dimers and transphosphorylation on its tyrosine residues, as well as the initiation of a cascade of downstream signaling pathways [3-7].

The role of HER4 in breast cancer remains controversial. In many studies, HER4 has been identified to have an anti-proliferative activity, which is contrary to other HER family members [8-10]. In addition, positive HER4 expression was shown to be associated with an increased survival rate of breast cancer patients [11]. A recent study conducted among HER2 positive breast cancer patients demonstrated that HER4 is predictive of longer event-free survival (EFS) in patients with ER positive tumours [12]. That study is in agreement with previous reports that linked high HER4 expression with low tumour grade, low proliferation rate and positive score for ER and PR in breast cancer [13-15]. However, there are also some studies correlated HER4 with increased cellular growth and oncogenic properties [16-18]. Bieche [19] reported that breast cancer patients with tumours overexpressing HER4 had a shorter survival rate, suggesting HER4 maybe an important biomarker for poor prognosis.

The role of HER4 in relation to trastuzumab treatment and resistance remains unclear. Although HER4 expression has been shown to improve trastuzumab response [20, 21], there has been no detailed report on how trastuzumab affects HER4 expression, activation and cleavage and how these processes mediate sensitivity and resistance to trastuzumab in HER2 positive breast cancer cells. The aim of this study is to elucidate the role and prognostic significance of HER4 expression and localisation in relation to trastuzumab treatment and resistance in HER2 positive breast cancer.

## RESULTS

### Trastuzumab treatment and exogenous heregulin stimulation induce the upregulation of HER4_180kDa_ and HER4_80kDa_ in HER2 positive breast cancer

We have previously reported that gefitinib and trastuzumab induce the endogenous release of heregulin in HER2 amplified breast cancer cells [22, 23]. In this study, we compared the effect of trastuzumab to exogenous heregulin stimulation on HER4 expression and activation in HER2 amplified SKBR3 and BT474 cells. MCF7 cells were used as the control cell line since they have a low level of HER2. The basal expression of HER4 in SKBR3 was comparable to the expression in MCF7, although it was lower in BT474 cells (Supplementary Figure 1A).

**Figure 1:**
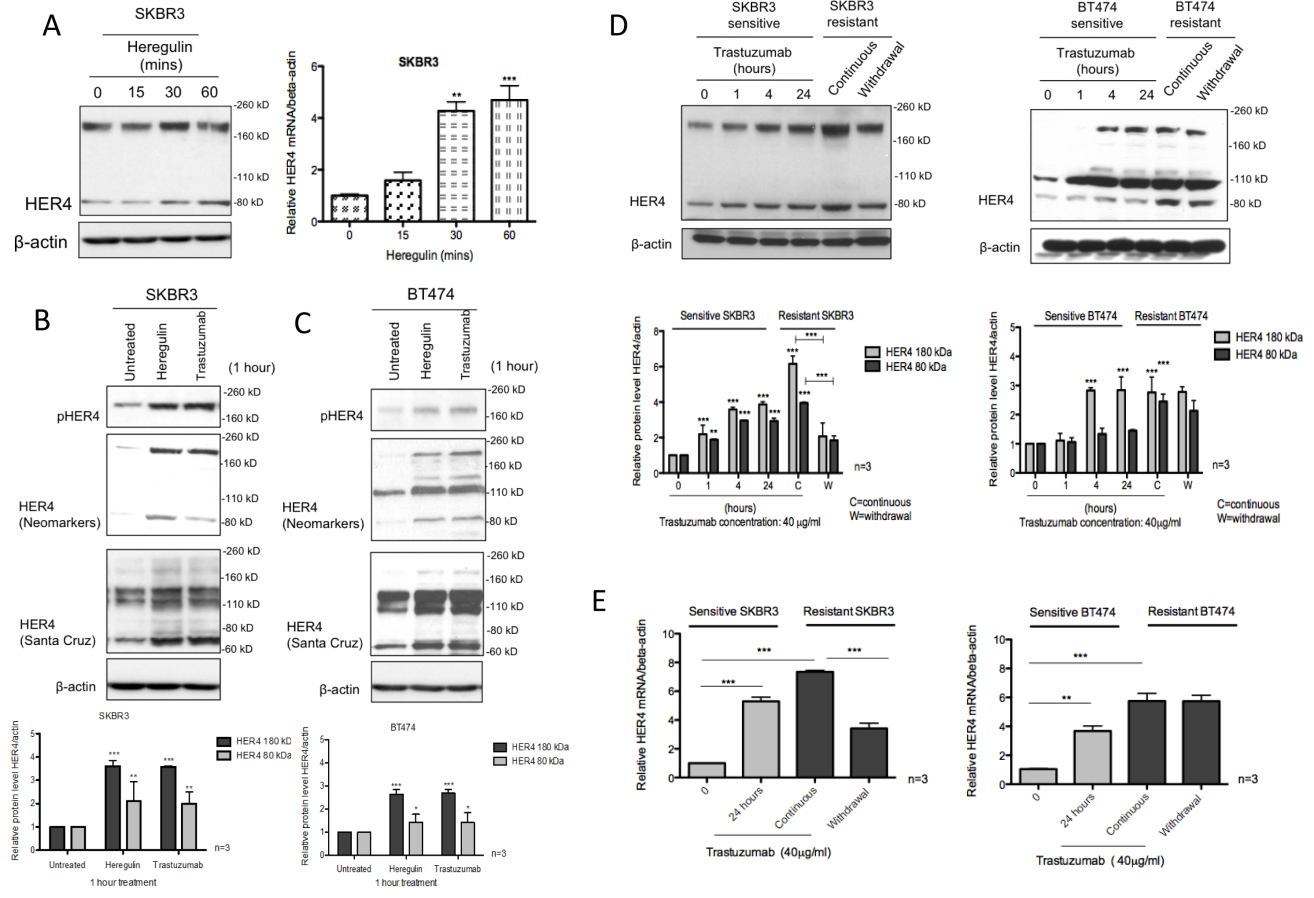
HER4 expression is upregulated following heregulin stimulation and trastuzumab treatment (A) The left panel shows the western blot of HER4 after 100ng/ml heregulin stimulation at different time points in SKBR3 cells. Right, SKBR3 cells were stimulated with 100ng/ml heregulin and HER4 expression was assessed by RT-PCR. HER4 mRNA of each group was quantified relative to the untreated cells, and normalised to β-actin. (B & C) SKBR3 and BT474 cells were treated with 40μg/ml trastuzumab or stimulated with 100ng/ml heregulin for one-hour before western blot analysis for the indicated proteins. Two different anti-HER4 antibodies, HER4 (Neomarkers) and HER4 C18 (Santa Cruz: SC283) were used. The quantification of HER4 protein level was done for anti-HER4 antibody from Neomarkers after normalised to β-actin (bottom panels). Means ± SD from three independent experiments are shown in the graph (*p<0.05, **p<0.01, ***p<0.001). (D) The figures show the representative western blots (upper panels) of HER4 and their quantification (lower panels) from sensitive SKBR3 and BT474 cells treated with 40μg/ml trastuzumab at 0, 1, 4 and 24 hours in comparison with trastuzumab-resistant cells (with or without trastuzumab 24 hr withdrawal). The resistant cell lines were previously generated by a continuous treatment with 40μg/ml trastuzumab for more than 8 months [23]. (E) HER4 mRNA levels of sensitive and trastuzumab resistant SKBR3 and BT474 cells were measured. β-actin mRNA was used for normalization and the quantification was relative to the untreated cells. The means of HER4 mRNA ± SD from three independent experiments are shown in the graph (**p<0.01, ***p<0.001).

Using two different antibodies of HER4 cytoplasmic domain, we showed that exogenous heregulin stimulation increased HER4 protein; and the mRNA levels were concordantly upregulated (Figure 1A-C). Similarly, exogenous heregulin stimulation or one-hour trastuzumab treatment resulted in an upregulation of HER4_180kDa_ and HER4_80kDa_ forms as well as an increase of HER4 phosphorylation in SKBR3 cells (Figure 1B). Using the Neomarkers' antibody to the cytoplasmic domain of HER4, western blots showed that there was also an increase of HER4 bands at 110 kDa and 120kDa in BT474 cells, which were not clearly seen in SKBR3 cells (Figure 1C). However, these two additional bands could be visualised using another anti-HER4 antibody from Santa Cruz. The immunoblot using these two different HER4 antibodies showed that the bands around 180 kDa, 110 kDa, 120 kDa and 80kDa (also known as m80) decreased following HER4 silencing, indicating that these are specific HER4 bands (Figure 3B). In HER2 negative MCF7 cells, trastuzumab treatment and heregulin stimulation also induced an upregulation of HER4 at 180kDa but no obvious increase in HER4_80kDa_ was detected (Supplementary Figure 1B). Since one-hour trastuzumab treatment upregulated HER4 expression, we next determined the dose effect of trastuzumab on HER4 at this time point. There was a dose responsive upregulation of HER4_80kDa_ from 10 to 20 μg/ml trastuzumab in SKBR3 and BT474 cells but no further increase beyond 20 μg/ml dose was seen (Supplementary Figure 1C). No obvious changes in HER2 level could be detected with increasing doses of trastuzumab treatment in SKBR3 and BT474 cells after 1 hour.

### HER4_180kDa_ and HER4_80kDa_ increase upon acquired resistance to trastuzumab

We also performed a time-course experiment to assess the effect of trastuzumab treatment on HER4 expression in SKBR3 and BT474 cells in comparison with the acquired resistant cells generated in the lab [23] (Supplementary Figure 1D). In the parental SKBR3 cells, trastuzumab treatment significantly increased HER4_180kDa_ and HER4_80kDa_ after a 24-hour treatment (n=3, p<0.01) and also in acquired resistant SKBR3 cells (n=3, p<0.001) (Figure 1D, left panel). Similar results were seen in the parental and resistant BT474 cells (Figure 1D, right panel). In contrast to trastuzumab-resistant cells that were continuously treated with trastuzumab, an overnight trastuzumab withdrawal reduced HER4_180kDa_ and HER4_80kDa_ significantly (n=3, p<0.001) in the trastuzumab-resistant SKBR3 cells but not in the resistant BT474 cells (Figure 1D). There was also a statistically significant increase of HER4 mRNA levels after trastuzumab treatment and upon acquired resistance (Figure 1E). Withdrawal of trastuzumab from the resistant SKBR3 cells decreased HER4 mRNA level (n=3, p<0.001) (Figure 1E, left panel), which was not seen in BT474 cells (Figure 1E, right panel).

### Heregulin stimulation and trastuzumab treatment induce HER4 nuclear translocation

**Figure 2:**
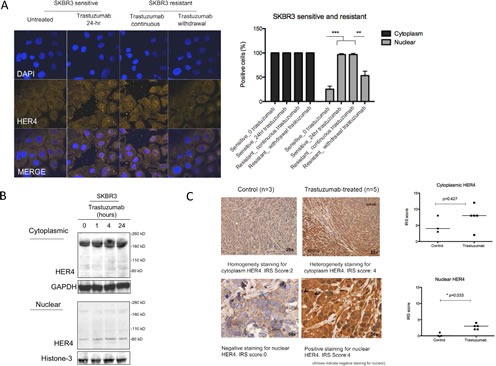
Nuclear HER4 localisation is induced *in vitro* and *in vivo* after trastuzumab treatment (A) Left, representative confocal images from sensitive and acquired resistant SKBR3 cells treated with 40μg/ml trastuzumab are shown. Right, a graph shows the percentage of positive cells stained with HER4-Alexa Fluor 546 at cytoplasmic and nuclear localisation. Means ± SD from three independent experiments are shown in the graph. 1-way ANOVA test with Bonferroni's multiple comparisons was applied to determined significant differences between the groups (**p<0.01, ***P<0.001). (B) Western blot analysis was performed on the cytoplasmic and nuclear fractions of SKBR3 cells isolated using NE-PER Nuclear and Cytoplasmic Extraction kit (Thermo Scientific). After cell fractionation, GAPDH and Histone-3 levels were used as the loading control for cytoplasm fraction and nuclear fraction respectively. (C) Left, HER4 staining by IHC in untreated and trastuzumab treated BT474 xenografts. The xenografts consist of mice treated with either trastuzumab or control for two weeks [24]. HER4 expression was scored semiquantitatively using the immunoreactive score (IRS) as described in the methods. Right, the cytoplasmic and nuclear HER4 scorings of trastuzumab-treated and the control groups are presented in a graph. Mann-Whitney test was used to determine the difference in median IRS scores between the untreated and trastuzumab-treated group. The statistical significance was denoted as * p<0.05.

We next assessed the effect of heregulin stimulation and trastuzumab treatment on HER4 localisation. In untreated parental SKBR3 cells, HER4 was localized to both cytoplasmic and nuclear compartments (Figure 2A). Heregulin stimulation (Supplementary Figure 2A) and trastuzumab treatment (Figure 2A) induced HER4 nuclear localisation in SKBR3 cells. Compared to the untreated SKBR3 cells, the percentage of the cells that had nuclear HER4 staining was increased at 24 hours after trastuzumab treatment (n=3, p<0.001) (Figure 2A, right panel). There was also increased basal HER4 nuclear translocation in acquired resistant SKBR3 cells (n=3, p<0.001) (Figure 2A, right panel) and resistant BT474 cells (n=3, p<0.05) (Supplementary Figure 2B). Trastuzumab withdrawal in trastuzumab resistant SKBR3 cells significantly decreased the percentage of positive nuclear HER4 cells (n=3, p<0.01) (Figure 2A, right panel). Cell fractional experiments showed that trastuzumab treatment upregulated HER4_180kDa_ but not HER4_80kDa_ in the cytoplasmic fraction (Figure 2B). However, there was an increased HER4_80kDa_ in the nuclear fraction, correlated with the HER4 localisation seen in confocal experiments (Figure 2B).

We also investigated an *in vivo* BT474 xenograft model. We first optimized HER4 immunohistochemistry (IHC) staining using a HER2 positive cell pellet (Supplementary Figure 2C). Figure 2C (left panels) shows an example each of HER4 IHC staining on BT474 xenograft tumour samples treated with either control or trastuzumab [24]. In the untreated group, HER4 cytoplasm was stained homogenously with no or minimal HER4 staining in the nucleus. Following trastuzumab treatment for two and half weeks, HER4 was increased at both cytoplasm and nucleus but only nuclear HER4 staining was statistically significant (median IRS score=3, n=3) compared to the control (median IRS score=0, n=5) (p<0.05) (Figure 2C, right panel). Taken together, the data shows that trastuzumab treatment and resistance induced HER4 nuclear translocation *in vitro*; and there was increased cytoplasmic and nuclear HER4 staining in BT474 xenograft models treated with trastuzumab.

### HER4 knockdown decreases cell viability and enhances trastuzumab response

**Figure 3:**
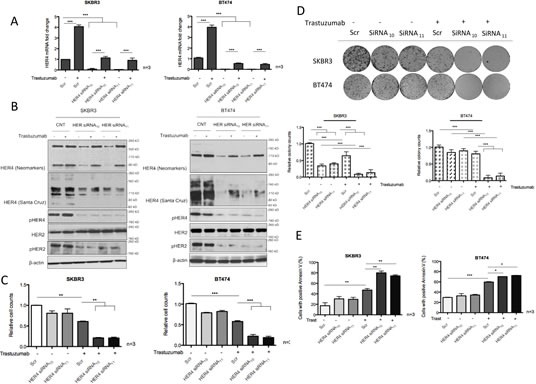
HER4 silencing decreases cell viability and enhances trastuzumab responses (A) SKBR3 and BT474 cells were transfected with HER4 siRNA_10_ and siRNA_11_, with and without 40μg/ml trastuzumab treatment before the mRNA levels were measured by RT-PCR. The scr (scramble) siRNA was used to normalize for transfection efficiency. Expression of HER4 is relative to the untreated scramble control and is normalised to β-actin. (B) Representative western blots of the indicated proteins from HER4 siRNA experiments in (A) are shown. (C) Cell count experiments were performed in SKBR3 and BT474 cells after 72-hour transfection as in (A) and the relative cell counts (normalized to the scr control) is shown in the graph. Means ± SD of three independent experiments is shown (**p<0.01 ***p<0.001). (D) The effect of HER4 siRNAs in SKBR3 and BT474 cells was further investigated in colony formation experiments after 7 days post transfection as in (A). Representative colony pictures are shown on the upper panels and the relative colony counts (means ± SD) are shown on the bottom panels. (E) SKBR3 and BT474 cells transfected with transient HER4 siRNA (with and without trastuzumab) for 72 hours were stained for Annexin V and the percentage of positive cells was assessed by FACS analysis. Means ± SD from three independent experiments are shown in the graph (*p<0.05, ***p<0.001).

To further understand the role of HER4 in trastuzumab-treated HER2 positive breast cancer cell lines, we performed HER4 knockdown by transient transfection with specific HER4 siRNAs, with or without trastuzumab treatment. Two independent HER4 siRNAS, HER4 siRNA_10_ and HER4 siRNA_11_ diminished HER4 mRNA and protein expression significantly in SKBR3 cells and BT474 cells (Figure 3A and 3B). The addition of trastuzumab increased HER4 mRNA and protein levels in the control siRNA treated cells (Figure 3A and 3B). Although HER4 silencing could not completely prevent trastuzumab-induced increase in the mRNA and protein levels of HER4, they were less than the control siRNA treated cells (Figure 3A and B). HER4 knockdown also lead to a decrease of HER4 phosphorylation in trastuzumab treated and untreated cells (Figure 3B). Although HER4 knockdown had no effect on HER2 level, there was a decrease in pHER2 level, indicating the dependency of HER2 activation on HER4.

We proceeded to assess the effect of HER4 knockdown with or without trastuzumab treatment using cell viability experiments in SKBR3 and BT474 (Figure 3C). Although HER4 knockdown alone has a minimal effect in decreasing the cell number and apoptosis in both cell lines, it had an inhibitory effect on tumour colony formation in SKBR3 cells (and not in BT474 cells) (Figure 3C-E). However, HER4 knockdown enhanced trastuzumab sensitivity significantly as shown by decreasing cell number and colony formation as well as increasing apoptosis in both SKBR3 and BT474 cells compared to the control cells (Figure 3C-E).

**Figure 4:**
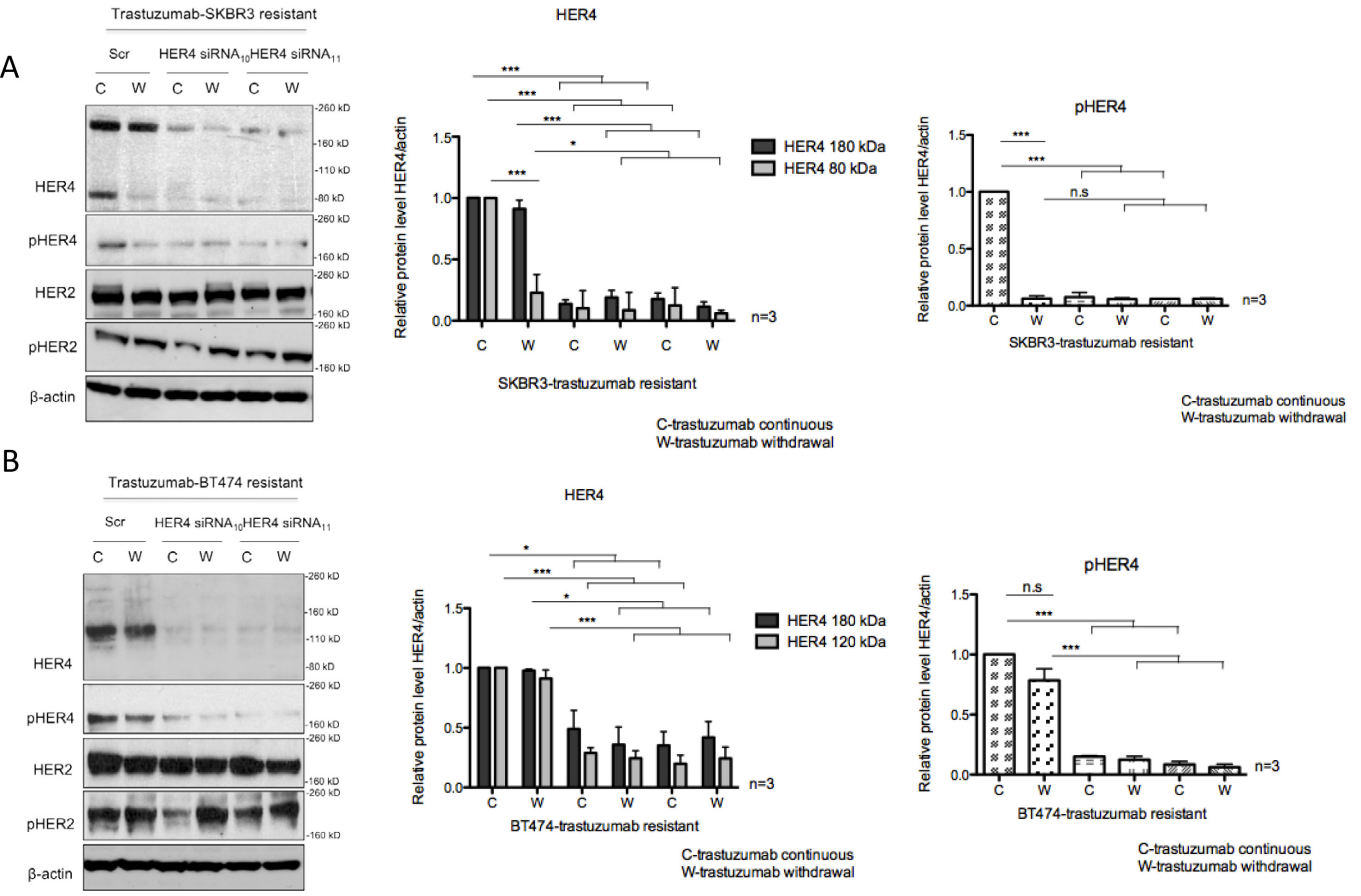

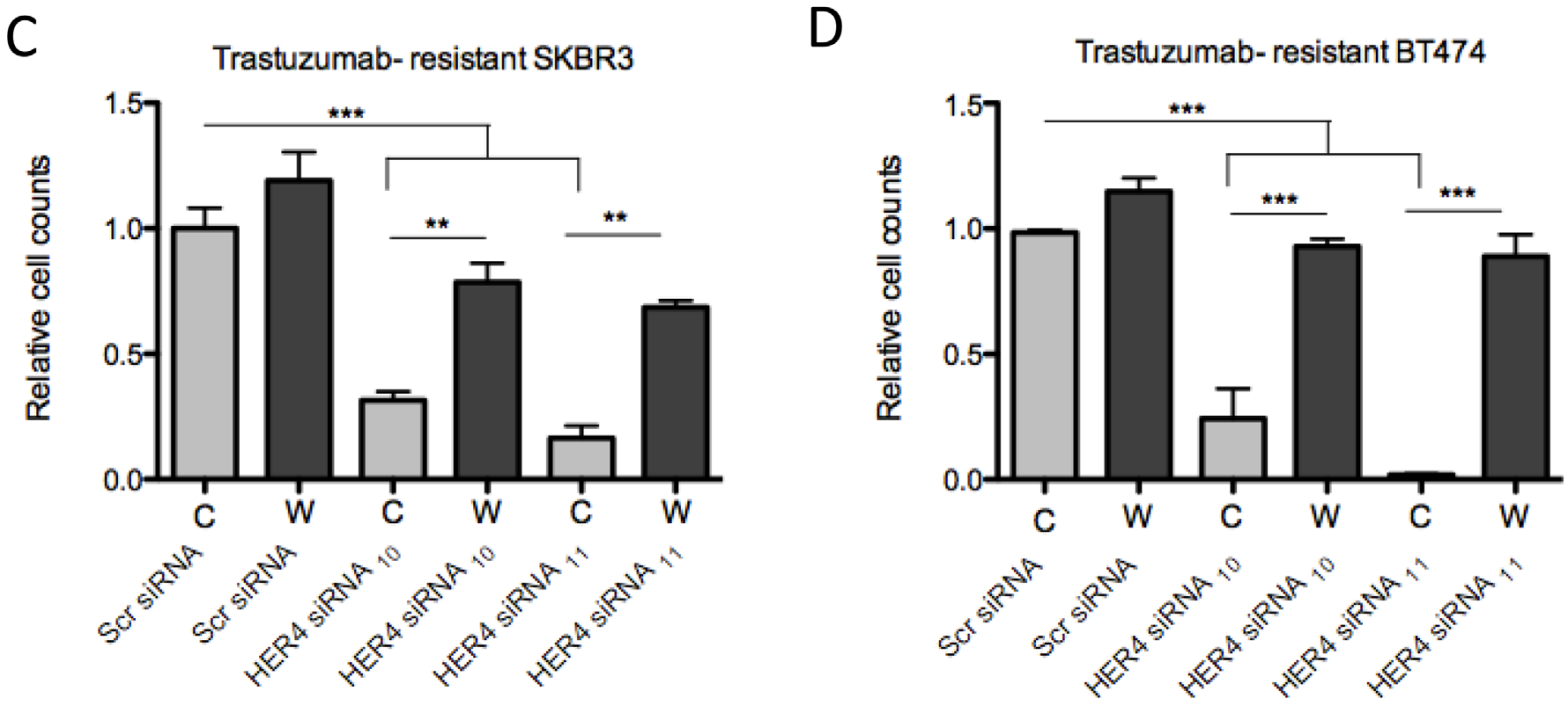
HER4 silencing reverses trastuzumab resistance and decreases cell counts in trastuzumab-resistant cells (A & B) Trastuzumab-resistant SKBR3 and BT474 cells were transfected with HER4 siRNA_10_ and siRNA_11_ in the presence (C) or withdrawal (W) of trastuzumab before western blot analysis for the indicated proteins. The middle and right panels show a relative quantification of HER4 and pHER4 levels from 3 independent experiments using Image J software. A 1-way ANOVA test with Bonferroni's multiple comparison was applied to determine significant differences between the groups (n.s=not significant, *p<0.05, ***p<0.001) (C & D) Cell count experiments were performed at 72 hr post transfection after siRNA experiments as in (A) and (B). The graphs show relative cell count relative to the scramble resistant cells. The mean ratios of cell counts ± SD from three independent experiments are shown (**p<0.01, ***p<0.001).

To further assess the role of HER4 in acquired resistance to trastzumab, HER4 was also silenced in trastuzumab resistant cells in the presence or absence of continuous trastuzumab treatment. Trastuzumab withdrawal decreased HER4 m80 and pHER4 in the resistant SKBR3 cells but not in the resistant BT474 cells (Figure 4A and 4B). HER4 and its phosphorylation also decreased after HER4 knockdown with and without combination treatment with trastuzumab (Figure 4A and 4B). Although total HER2 level remained unchanged, pHER2 level increased when trastuzumab was withdrawn from trastuzumab-resistant cells transfected with either the control or HER4 siRNAs (Figure 4A and 4B).

HER4 knockdown had an inhibitory effect on the cell count and reversed trastuzumab resistance in both resistant cell lines continuously treated with trastuzumab (Figure 4C and 4D). However, trastuzumab withdrawal diminished the inhibitory effect of HER4 knockdown in the resistant cells (Figure 4C and 4D).

### γ-secretase inhibitor decreases HER4 cleavage and nuclear translocation as well as enhancing trastuzumab sensitivity

**Figure 5:**
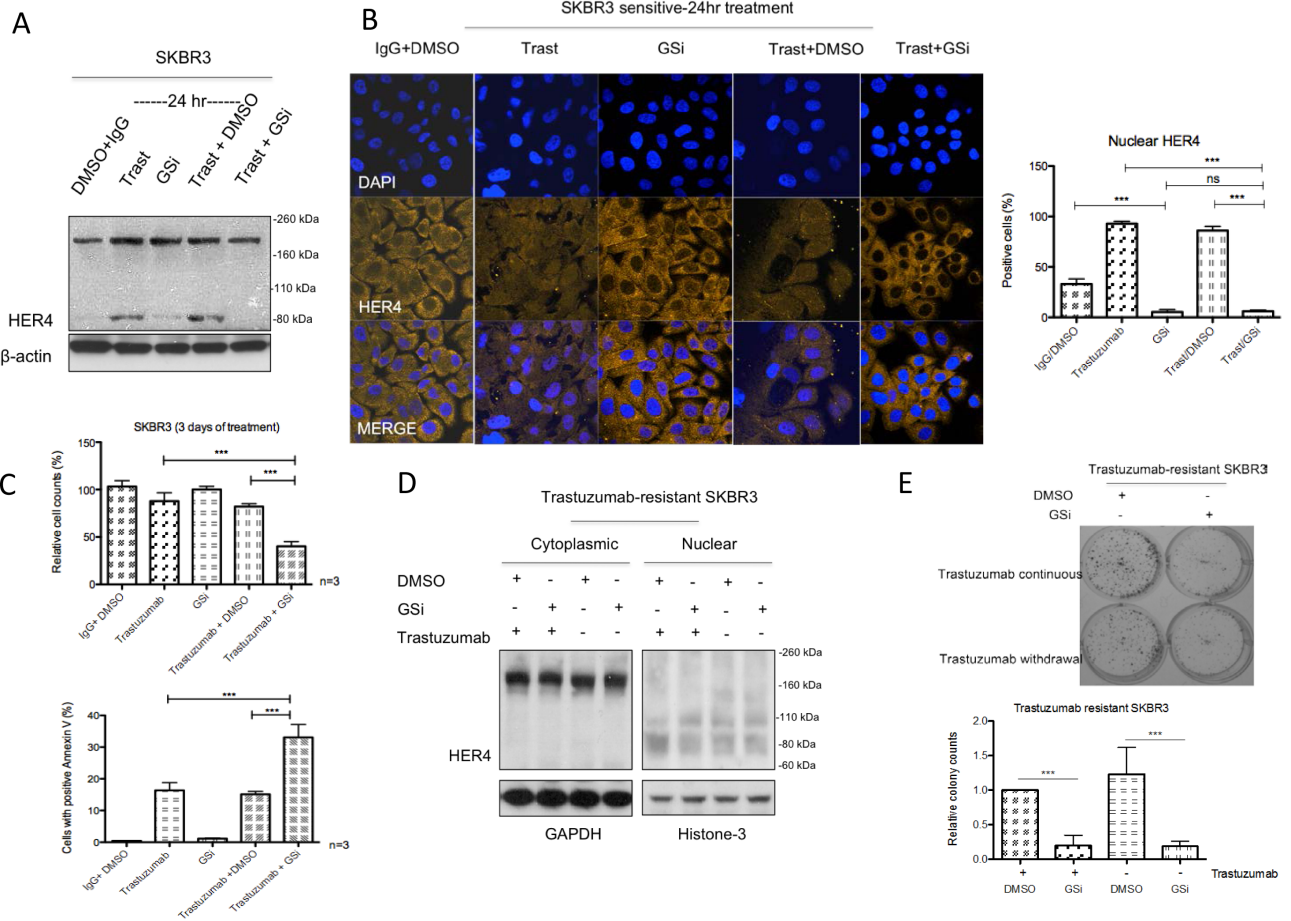
Gamma-secretase inhibitor prevents nuclear translocation and reverses resistance in trastuzumab-resistant cells (A & B) SKBR3 cells were treated with 10μM γ-secretase inhibitor (GSi) with or without 40μg/ml trastuzumab before performing western blot analysis and confocal microscopy to investigate HER4 expression by IHC and localization by confocal microscopy. (C) Cell count and apoptosis assays were performed after treatments as in (A) and (B). Means ± SD from three independent experiments are shown (***p<0.001). (D) Figures show the representative HER4 immunoblots on cytoplasmic and nuclear fractions from trastuzumab-resistant SKBR3 cells treated with GSi. (E) The effect of GSi on trastuzumab-resistant SKBR3 was studied using colony formation assay. Representative colony pictures are shown on the upper panel and the relative ratio of colony counts are shown on the bottom panel.

Gamma-secretase (γ-secretase) is involved in the proteolytic cleavage of transmembrane HER4 following the initial release of extracellular domain by ADAM17, resulting in the production of cleaved HER4_80kDa_ and its nuclear translocation [25, 26]. Since we have shown that trastuzumab increased HER4_80kDa_ and induced its nuclear translocation in HER2 positive breast cancer (Figure 2A and 2B), we tested whether a γ-secretase inhibitor (GSi) could prevent HER4 nuclear localisation induced by trastuzumab treatment. Although trastuzumab treatment increased HER4_80kDa_ (Figure 5A), the combination treatment of trastuzumab with GSi in SKBR3 prevented the increase. Figure 5B (left panel) shows the confocal images from SKBR3 treated with GSi, in the presence or absence of trastuzumab treatment for 24 hour. GSi alone decreased HER4 intensity in the nucleus compared to the control (n=3, p<0.001) (Figure 5B). In addition, Figure 5B also shows that when GSi was added to trastuzumab, not only did it decrease trastuzumab induced nuclear HER4 enhancement, but there was also an upregulation of cytoplasmic HER4 intensity. The decreased of the percentage of positive nuclear HER4 staining in the combination treatment of GSi and trastuzumab was statistically significant compared to trastuzumab alone or trastuzumab with DMSO (n=3, p<0.001) (Figure 5B, right panel). After 3 days of treatment, trastuzumab in combination with GSi resulted in a significant decrease in cell viability of SKBR3 cells compared to trastuzumab alone or GSI alone (Figure 5C, upper panel). There were also a significant increase in the number of apoptotic SKBR3 cells, when treated with the combination of GSi and trastuzumab, as compared to trastuzumab alone or GSi alone (Figure 5C, bottom panel). In trastuzumab-resistant SKBR3 cells continuously treated with trastuzumab, GSi decreased HER4_80kDa_ in the nucleus (Figure 5D). Trastuzumab withdrawal resulted in a decrease in the nuclear HER4_80_ but GSi did not further change its level in resistant SKBR3 cells. Trastuzumab withdrawal in the trastuzumab-resistant SKBR3 cells did not show any significant effect on colony formation. However, GSi decreased colony formation in resistant cells with or without trastuzumab treatment (Figure 5E).

### Neratinib prevented trastuzumab-induced upregulation of HER4 in the cytoplasm and the nucleus

**Figure 6:**
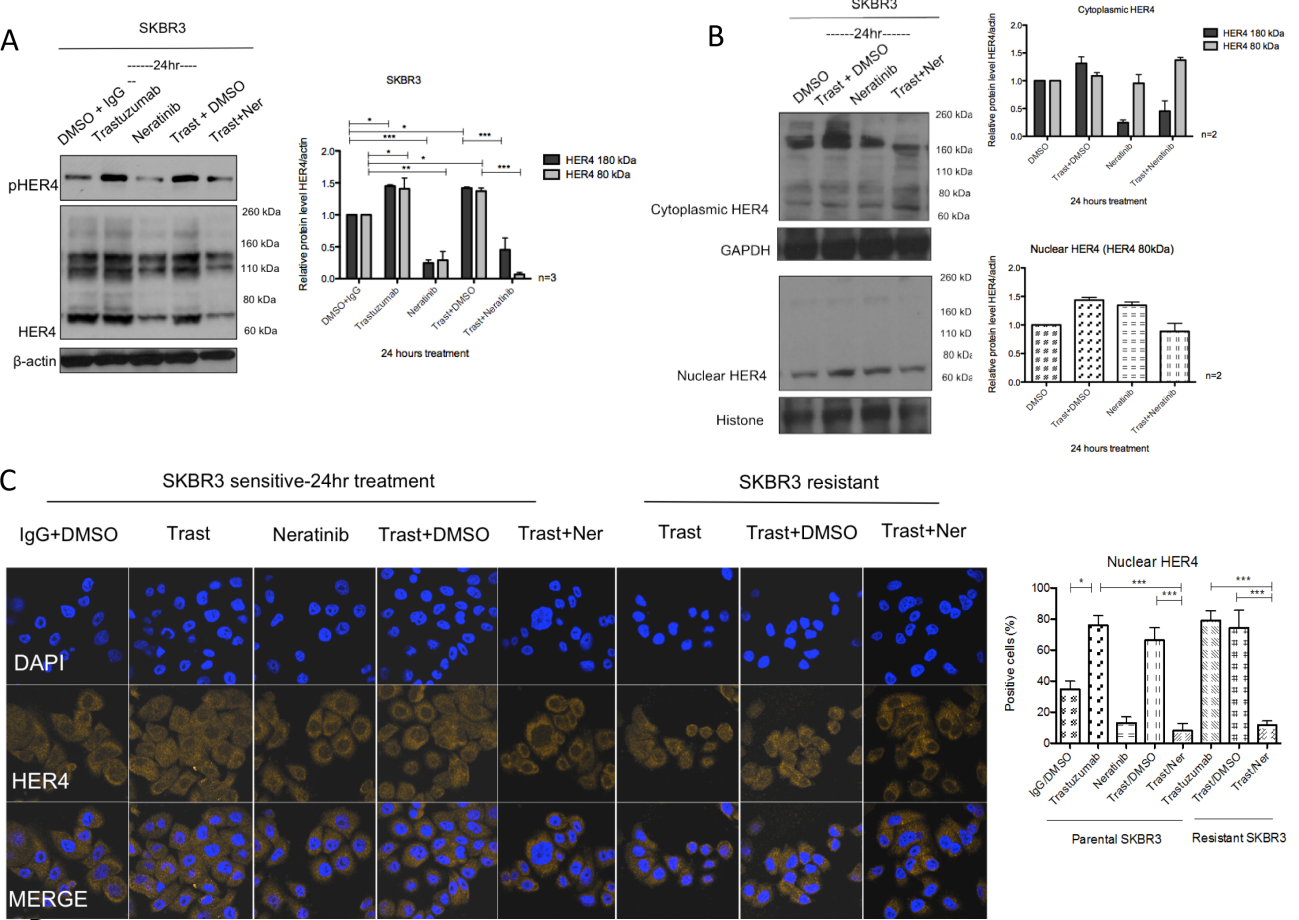

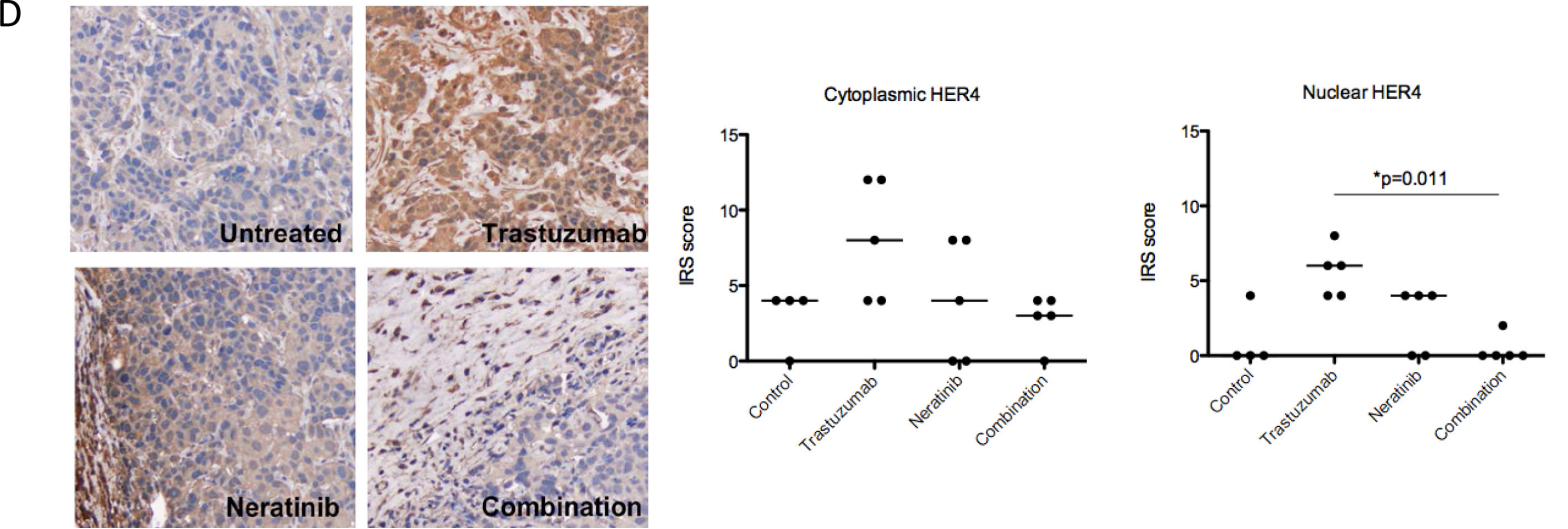
Neratinib prevents nuclear HER4 translocation induced by trastuzumab *in vitro* and *in vivo* (A) SKBR3 cells were treated with 100 nM neratinib or/and 40μg/ml trastuzumab for 24 hours before western blot analysis for the indicated proteins. (B) Western blot analysis was performed on the cytoplasmic and nuclear fractions of SKBR3 cells after treatments in (A). On the left panels, the representative blots are shown. The right panels show a relative quantification of HER4 and pHER4 from 2 independent experiments using Image J software. Although multiple independent experiments were done, only two experiments provided adequate quality of HER4 immunoblot for quantification. (C) Sensitive and resistant SKBR3 cells were treated with 100 nM neratinib and/or 40μg/ml trastuzumab before being fixed for confocal microscopy. Representative confocal images from different treatment conditions are shown on the upper panel. The right panel shows the percentage of positive cells stained with HER4-Alexa Fluor 546 at nuclear localisation. Means ± SD from three independent experiments are shown in the graph. 1-way ANOVA test with Bonferroni's multiple comparisons was applied to determined significant differences between the groups (*p<0.05, ***p<0.001).(D) BT474 xenografts were treated with vehicle control, neratinib, trastuzumab or their combination [28] and the tumour slides were stained for HER4 expression using IHC. Representative tumour sections from formalin-fixed, paraffin embedded tumour tissues of animals receiving different treatments are shown in the left panels. The IRS scores of HER4 immunohistochemical staining of cytoplasmic and nuclear localisation of the tumours are shown in the right panels. Kruskal-Wallis with Dunn's multiple comparisons test was used to determine the difference in median IRS scores between the treatment groups (*P<0.05).

Neratinib is an irreversible TKI targeting tyrosine kinase activities of HER1, HER2 and HER4 [27]. We have previously reported that neratinib enhances trastuzumab response and is effective in overcoming the acquired resistance of trastuzumab [28]. However, its effect on HER4 cleavage and localisation was not investigated. Since HER4 cleavage is induced upon its activation [3-7], we proceeded to assess the effect of neratinib on HER4 deactivation as well as its expression and localisation in HER2 positive breast cancer cells. As expected, neratinib decreased HER4 tyrosine kinase phosphorylation in SKBR3 cells (Figure 6A). Neratinib treatment (in the presence or absence of trastuzumab) led to a decrease in both 180 kDa and 80 kDa of HER4 in the total cell lysate of SKBR3 cells (Figure 6A). In the parental and resistant BT474 cells, neratinib also decreased the intensity of several HER4 bands around 180kDa, 110kDa and 80kDa with and without trastuzumab in the total cell lysate (Supplementary Figure 3A).

We also performed cell fractionation experiments and showed that neratinib prevented trastuzumab-induced increase of HER4_80kDa_ in the nucleus after 24h neratinib treatment in SKBR3 cells. However, neratinib alone did not decrease m80 in the nucleus (Figure 6B). Confocal images also showed that there was a decrease in the percentage of cells with positive HER4 staining in the sensitive and resistant SKBR3 cells treated with a combination treatment of trastuzumab and neratinib, as compared to trastuzumab treatment alone (both n=3, p<0.001) (Figure 6C). These results correlated with a greater inhibitory effect of the combination treatment of neratinib with trastuzumab compared to trastuzumab alone on colony formation in both sensitive and resistant SKBR3 cells (Supplementary Figure 3B).

In BT474 xenograft models treated with trastuzumab and/or neratinib [28], there was an increase in cytoplasmic and nuclear HER4 staining in xenograft tumour samples treated with trastuzumab. There was no difference in the nuclear and cytoplasmic HER4 scorings between those treated with neratinib and the control. However, trastuzumab-induced HER4 upregulation was prevented by the combination treatment of trastuzumab with neratinib (Figure 6D). The decrease in nuclear HER4 scoring was statistically significant in the combination treatment of trastuzumab and neratinib (median IRS score=0, n=5) compared to trastuzumab alone (median IRS score=6, n=5) (p<0.05) (Figure 6D, right panel).

### Nuclear HER4 is a prognostic factor in HER2 positive breast cancer patients

**Figure 7:**
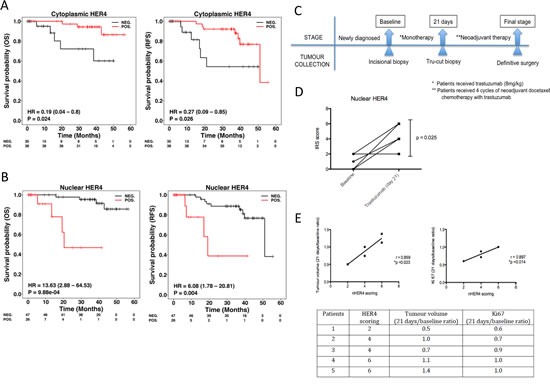
Nuclear HER4 predicts poorer trastuzumab response and is an adverse prognostic marker in HER2 positive breast cancer patients (A & B) TMAs containing paraffin embedded breast tumours from a well-annotated HER2 positive breast cancer patients were stained for HER4 expression by IHC. Patients were split into two groups according to the positive and negative cytoplasmic and nuclear HER4 staining as defined in the methods section. The relapse-free survival and overall survival of the two groups were plotted in Kaplan-Meier curves according to the cytoplasmic and nuclear HER4 staining in breast cancer patients (n=73). (C) A schematic diagram shows a window of opportunity study conducted among HER2 positive breast cancer patients who received trastuzumab monotherapy followed by neoadjuvant chemotherapy with trastuzumab before surgery. Tumour specimens were collected at the baseline, 21 days after trastuzumab monotherapy and at the final stage after completed 4 cycles of neoadjuvant docetaxel chemotherapy 100 mg/m^2^ with 6mg/kg trastuzumab. (D) Nuclear HER4 expression was analysed using IHC in paired tissue samples (baseline and at 21 days) from 5 patients who received one dose of trastuzumab monotherapy. The differences in HER4 IRS scores between the pairs were asssesed by paired t-test (* indicates p<0.05). (E) Nuclear HER4 expression at day 21 was correlated with the ratios of tumour volumes and Ki67 stainings (between day 21 and baseline) using linear regression (upper panels). The table below shows the HER4 scorings of the individual 5 patients with their respective tumour volume and Ki67 ratios.

To assess the prognostic value of HER4 localisation, we stained TMAs from a well-annotated cohort of HER2 positive breast cancer patients for HER4 expression. The characteristics of the patients and tumour parameters are listed in Supplementary Table 1. The results showed that high cytoplasmic HER4 was significantly correlated with better overall survival (OS) and relapse-free survival (RFS) than that of patients with low cytoplasmic HER4 (OS: HR = 0.19, 95% CI = 0.04−0.80, p = 0.024; RFS: HR = 0.27, 95% CI = 0.09−0.85, p = 0.026) (Figure 7A). In contrast, Kaplan-meier analysis of nuclear HER4 expression demonstrated an inverse correlation between positive nuclear HER4 expression and patient survival (IRS score ≥ 1 vs IRS score = 0; OS: HR = 13.63, 95%CI = 2.88−64.53, p < 0.001; RFS: HR = 6.08, 95%CI = 1.78−20.81, p = 0.004) (Figure 7B). In a multivariate analysis with nuclear HER4-derived groups adjusted for ER and nodal status, nuclear HER4 remained an independent prognostic factor for OS (p = 0.002) and RFS (p = 0.011); whereas cytoplasmic HER4 appeared to be a weak prognostic predictor of OS (p = 0.059) and RFS (p = 0.058) (Supplementary Table 2).

### Nuclear HER4 is predictive of trastuzumab response in HER2 positive breast cancer patients

We also assessed HER4 expression in HER2 positive breast cancer patients who received trastuzumab monotherapy followed by neoadjuvant chemotherapy with trastuzumab treatment (Figure 7C). The patient characteristics and tumour parameters are listed in Supplementary Table 3. There were 5 out of 10 patients underwent biopsy before and after trastuzumab monotherapy treatment. After trastuzumab monotherapy, there was a statistically significant upregulation of nuclear HER4 expression in the tumour biopsies collected at day 21 compared to the biopsies from the initial diagnosis (mean HER4 IRS at day 21=3.20, 95%CI = 1.84−4.56 vs. mean HER4 IRS at baseline=0.80, 95%CI=−0.56−2.16, p=0.025) (Figure 7D). In addition, higher nuclear HER4 expression at day 21 was correlated with a poorer clinical response to trastuzumab monotherapy, including a higher tumour volume (r=0.859, p=0.023) and Ki67 level ratios (r=0.897, p=0.014) (Figure 7E). However, there was no statistically significant difference in the tumour nuclear HER4 expression between baseline and after neoadjuvant chemo with trastuzumab (Supplementary Figure 4A). In addition, nuclear HER4 after neoadjuvant chemotherapy did not correlate with the tumour volumes and Ki67 values obtained from definitive surgery (Supplementary Figure 4B).

## DISCUSSION

In this study, we showed that trastuzumab treatment upregulated HER4 at 180 kDa and 80 kDa in SKBR3 and BT474 cells, the common HER4 variants reported from the previous study [29, 30]. HER4_80kDa_ has been described as a cleaved fragment of HER4 and is known to translocate to the nucleus [8, 31-33]. Our study demonstrates that trastuzumab-induced HER4_80kDa_ correlated with increased HER4 nuclear intensity from confocal images and increased cleaved HER4 fragment (HER4_80kDa_) in the nuclear fraction of trastuzumab treated cells. The xenograft experiments also showed that nuclear HER4 staining was significantly increased in the tumours of the mice treated with trastuzumab compared to the untreated group. Furthermore, trastuzumab resistant SKBR3 and BT474 cells showed an increased intensity of nuclear HER4 compared to the sensitive cells. These results suggest that HER4 cleavage and nuclear translocation occurs during trastuzumab treatment and resistance.

Depending on the antibodies used and in different cell lines (Figure 1B and 1C), we also observed the appearance of multiple HER4 bands at 180kDa, 120kDa, 110 kDa and 80 kDa. To our knowledge, no study has reported HER4 variants at 110 and 120 kDa. However, these bands were confirmed to be specific to be HER4 by the siRNA experiments (Figure 3B). From the recent publications, other HER receptors were also reported to have multiple variants, including HER2 at 185, 150, 110 and 95 kDa of molecular weight [34, 35] and multiple HER3 variants in Schwann cell model [36]. We therefore suggest that a future study could be carried out to characterize HER4 variants at 110 and 120 kDa to assess whether they could represent different HER4 isoforms or alternative cleaved fragments.

Although HER4 has been shown to have an anti-proliferative activity in breast cancer cells [8-10], we showed that HER4 silencing decreased cell viability and enhanced trastuzumab effect as well as reversed its resistance in HER2 positive breast cancer cells. Our study suggested that during trastuzumab treatment, HER4 may mediate trastuzumab resistance and reduces its inhibitory effect. This seems to contradict other studies [20, 21]. However, the controversial effect of HER4 may be related to its cellular localisation. HER4 has been shown to translocate to the nucleus following its cleavage and interact with different transcription factors [32, 37-41]. Since trastuzumab induces the release of endogenous heregulin [23] and this could induce γ-secretase-mediated HER4 cleavage and nuclear localisation [26], we utilized γ-secretase inhibitor and showed that it decreased m80 and nuclear HER4 translocation in HER2 positive breast cancer cells when combined with trastuzumab. This was correlated with an increase in apoptosis and decreased cell proliferation, consistent with reports that γ-secretase inhibitor could inhibit heregulin-dependent tumour growth [26, 42]. Our study provides evidence that HER4 nuclear translocation during trastuzumab treatment is due to its proteolytic cleavage mediated by γ-secretase. However, we could not exclude that the growth inhibition by γ-secretase inhibitor could also be due to its effect on other signaling pathways, e.g. Notch or E-cadherin [43, 44].

There has been a recent study investigating the combination effect of a ganmma-secretase inhibitor in combination with docetaxel chemotherapy in breast tumorgraft models and in patients with advanced breast cancer [44]. The combination of both drugs resulted in a preliminary evidence of efficacy in patients (11 partial response out of 24 evaluable patients). However, this study used gamma-secretase inhibitor to inhibit Notch signaling and stem cell renewal but not HER4 cleavage. To our knowledge, we are not aware of any study that combines trastuzumab with gamma-secretase inhibitor to target HER4 cleavage and nuclear localization in HER2 positive breast cancer. It is hoped that our study may generate interest in targeting HER4 cleavage using gamma-secretase inhibitor in HER2 positive breast cancer and possibly other cancers.

To assess whether HER4 cleavage and nuclear translocation during trastuzumab is due to its tyrosine kinase activity, we utilized neratinib, which is an irreversible panHER inhibitor that has an effect against EGFR, HER2 and HER4 tyrosine kinase activity [45, 46]. We showed that whereas trastuzumab increased m80 and HER4 nuclear translocation, neratinib could prevent the induction of m80 and HER4 nuclear translocation induced by trastuzumab. This highlights the importance in understanding the molecular mechanisms of actions and resistance between different anti-HER2 therapies. The different effects of neratinib and trastuzumab on HER4 could be one of the reasons why their combination was more effective than either drug alone in preclinical models [28].

The prognostic value of HER4 expression in relation to breast cancer survival has been controversial and could be attributed to different HER4 isoforms and its localization [11, 13, 17, 47-49]. Four different HER4 mRNA spliced isoforms known as JM-a/CYT1, JM-a/CYT2, JM-b/CYT1 and JM-b/CYT2 have been reported, which are derived from the alternative HER4 mRNA splicing [30, 50, 51]. In contrast to JM-b variant, the JM-a region consists of Adam-17 (TACE) cleavage site that allows initial process of HER4 proteolytic cleavage to produce the extracellular domain (ECD) and intracellular domain (ICD) [25, 29, 52]. This is followed by further cleavage of HER4 intracellular domain by the γ-secretase at the transmembrane domain, resulting in the nuclear translocation of HER4 [26].

A recent study of HER4 isoform expression in breast cancer demonstrated a correlation between JM-a isoforms (CYT1 and CYT2) with longer patients' survival [12]. In addition, studies have shown that cytoplasmic HER4 is linked with improved survival of breast cancer patients [12, 47]. In our cohort study, we also demonstrated a similar pattern that high level of cytoplasmic HER4 had a better prognosis in HER2 positive breast cancer patients. However, positive nuclear HER4 was correlated with poorer outcomes in HER2 positive breast cancer patients, which is in agreement with Juntilla' study [17]. These suggested that the different reports of HER4 on breast cancer survival might be due to failure to look at clinical significance of HER4 localisation in these patients.

HER4 expression has been reported to be associated with increased sensitivity to trastuzumab in patients with metastatic HER2 positive breast cancer [21]. Although HER2 and HER4 dimerization appeared to be oncogenic [53], a recent study showed that patients with HER2/HER4 co-over-expression had a better outcome after neo-adjuvant trastuzumab therapy and adjuvant trastuzumab therapy [20]. However, these studies did not directly assess HER4 expression in pre and post-trastuzumab treatment samples or assess the relation of nuclear HER4 to the response to trastuzumab monotherapy and prognosis of patients. Although we only had a small number of patients in our study, we have demonstrated that trastuzumab induced nuclear HER4 upregulation and that nuclear HER4 correlated with poorer trastuzumab response as well as shorter survival in HER2 positive patients. It will be important to further assess the significance of HER2/HER4 dimerization in relation to trastuzumab treatment and prognosis of HER2 positive breast cancer patients using validated HER dimerization assays [54].

In summary, this study provides a first report on the effect of trastuzumab treatment in HER4 cleavage and its nuclear translocation in HER2 positive breast cancer. Downregulation of HER4 and strategies to inhibit HER4 cleavage and nuclear translocation (by neratinib and γ-secretase inhibitor) enhances trastuzumab response and reverses its resistance. Furthermore, we showed that nuclear HER4 is an adverse prognostic factor and may predict poorer trastuzumab response in HER2 positive breast cancer. Further validation is required in large independent randomized trial to further determine the prognostic and predictive role of HER4 in relation to anti-HER2 treatment in breast cancer patients.

## MATERIALS AND METHODS

### Cells culture

MCF-7, BT474 and SKBR3 cell lines were provided by cell services lab at Cancer Research UK (Lincoln's Inn Fields laboratory), which has a stringent quality control in cell authenticity and has incorporated short-tandem repeat (STR) profiling for cell line validation. Trastuzumab resistant cell lines were generated as previously reported [22, 23, 28]. The MCF7, SKBR3, and trastuzumab-resistant SKBR3 cell lines were grown in DMEM containing 10% FCS and 10% FBS and with 100 IU/ml Penicillin, 100 mg/ml Streptomycin (PS). The BT474 and trastuzumab-resistant BT474 cell lines were maintained in RPMI 1640 containing 10% FCS and the same concentration of PS. All cells were grown in a humidified incubator at 37°C with 5% CO^2^. Routinely, the cells were sub-cultured once a week and the medium was changed for three times per week.

### HER4 transient transfection

The cells were transfected with specific interfering RNAs for HER4 (Qiagen) followed by trastuzumab treatment. The transfection reagent in a final volume of 2 ml/well were prepared an hour before being added onto the cells, which comprising the final volume of 20nM HER4 siRNA, the Oligofectamine and the Optimem serum-free medium. HER4 expression was determined at 72-hour post transfection in both protein and mRNA levels. To determine the siRNA HER4 efficiency, scramble siRNA (All Star Negative siRNA) was used as a control.

### Analysis of HER4 expression

Western blotting and RT-PCR were performed to demonstrate the expression of HER4 protein and mRNA. Protein lysates preparation and western blot procedure were done according to a standard protocol described previously [23]. For western blot, the antibodies against c-terminus HER4 (Santa Cruz), c-terminus HER4 (Neomarkers), phospho-HER4 (Santa Cruz, CA) were used.

Total RNA was harvested from the cell lines with Total RNeasy Mini Kit (Qiagen). Total RNA (10 ng) was reverse transcribed using the iScript DNA synthesis kit, with a melting temperature of 62°C. Quantitative RT-PCR assays were performed using standard SYBR-Green protocol. The following primers were used: HER4, forward 5'-TGT GAG AAG ATG GAA GAT GGC-3', reverse 5'-GTT GTG GTA AAG TGG AAT GGC-3', β-actin, forward 5'-GGCGGCACCACCATGTACCCT-3', reverse 5'-AGGGGCCGGACTCGTCATACT-3'. The exponential route of C_T_ value was converted into a linear form using the 2 ^−ΔΔCT^-relative calculation methods. The β-actin was used as the relative control.

### Cellular fractionation

The NE-PER Nuclear and Cytoplasmic Extraction kit (Thermo Scientific) was used to obtain cytoplasmic and nuclear extracts from the experimental cell lines, according to manufacturer's protocol. Both of the cytoplasmic and nuclear extracts were then prepared as loading samples for western blot analysis of HER4.

### Confocal microscopy

The immunofluorescence staining for HER4 was performed to assess HER4 localisation in relation to trastuzumab treatment and resistance. Approximately 10,000 of cells were seeded onto coverslips in 24-wells plate, a day before cell treatment. After treatment, the medium containing drugs were discarded and the cells were rinsed with PBS twice. The cells were incubated with fixation reagents; 4% PFA, Triton-X and Natrium Borohydrate, before blocking with 3% BSA. HER4 primary antibody was incubated for 3 hours and a subsequent incubation with Alexa-546 conjugated secondary antibody (rabbit) for 1 hour. The coverslips holding the treated cells was than mounted onto a microscope slide using the DAPI Fluoromount-G. The slides were visualized using the confocal microscopy.

### Cell count experiments

Prior to drug treatment or siRNA transfection, approximately 10,000 cells were plated in 24-wells plate a day before. The cells were then treated with the indicated drug(s) or conditions for 3 or 6 days. On the day of experiment, the treated cells were washed in PBS and harvested with trypsin before cell counting using the Coultier counter.

### FACS analysis

To assess apoptosis, the cells were stained with 5 μl annexin-V-fluorescein isothiocyanate (FITC) and 5 μl of propidium iodide (PI). The analysis was run using a flow cytometry detector, FACS Calibur (Becton Dickinson). The apoptotic cells were analysed at the early stage (annexin-V-positive, PI-negative) and the late apoptotic stage (annexin-V-positive, PI-positive).

### Xenograft studies

The animal experiments have been described in previous publications for trastuzumab treatment [24] or trastuzumab with neratinib treatment [28].

### Human tissue samples

Tissue microarrays (TMA) of HER2 positive breast cancer patients were obtained from Oxford Radcliffe Biobank. The use of TMAs complies with the Human Tissue Act 2004 of UK. Patients' characteristics and histology including age, sex, tumour grade, menopausal states, lymph node(s) and ER status as well as patient deaths and recurrences were obtained from the medical records.

A set of formalin-fixed paraffin embedded tissues was obtained from a clinical trial conducted at UOM Patologia Mammaria-Az. Instituti Ospitalieri di Cremona. This study was approved by local ethical approval (Protocol CE-21392/2012). This study included consecutive cases of HER2 positive breast cancer patients that underwent trastuzumab window study followed by neoadjuvant chemotherapy and trastuzumab treatment, which was performed from April 2010 to December 2012. Patients' clinical information was obtained from accessible medical records.

### HER4 immunostaining

The IHC technique was performed to assess HER4 expression in xenograft tumours and patients' tumours. HER4 expression level was scored semiquantitatively using the immunoreactive score (IRS). The final IRS score was determined by multiplying the intensity score (3, strong; 2, moderate; 1, mild; and 0, no staining), with the scoring of the percentage of positive cells (4, >80%; 3, 51–80%; 2, 10–50%; 1, <10%; 0, 0%), giving the scoring between 0 (no staining) and 12 (maximum score). HER4 staining was catagorized into negative cytoplasmic HER4 (IRS <6) or positive cytoplasmic HER4 (IRS≥6); negative nuclear HER4 (IRS=0) or positive nuclear HER4 (IRS≥1) staining.

### Statistical Analysis

The data analysis was done using the GraphPad Prism 6 for MAC OS X. For *in vitro* experiments, statistical analyses were performed using t-Test for a comparison between two groups and one-way ANOVA with Bonferonni's multiple comparison test for a comparison among multiple groups. Data were expressed as means ± SD. In *in vivo* models, the Mann-Whitney test was used to compare the medians of HER4 protein level between two groups while the Kruskal Wallis with Dunn's multiple comparison test was applied on the analysis involving more than two groups. The Kaplan-Meier survival curves analysis and the multivariate Cox proportional hazards modeling were carried out in R statistical environment (v.2.14.1) (R package: survival v2.36-14), to correlate HER4 scorings with disease-free and overall survivals. In addition, the correlation of HER4 scorings with the other clinical parameters was assessed using Fisher's Exact test. For all statistical analysis, P values of < 0.05, <0.01 and <0.001 were considered as statistically significant and were marked as *, ** and *** respectively.

## SUPPLEMENTARY MATERIAL TABLES AND FIGURES



## References

[1] Muraoka-Cook RS, Feng SM, Strunk KE, Earp HS (2008). ErbB4/HER4: role in mammary gland development, differentiation and growth inhibition. J Mammary Gland Biol Neoplasia.

[2] Koutras AK, Fountzilas G, Kalogeras KT, Starakis I, Iconomou G, Kalofonos HP (2010). The upgraded role of HER3 and HER4 receptors in breast cancer. Crit Rev Oncol Hematol.

[3] Carpenter G (2003). ErbB-4: mechanism of action and biology. Exp Cell Res.

[4] Zhou W, Carpenter G (2000). Heregulin-dependent trafficking and cleavage of ErbB-4. J Biol Chem.

[5] Carraway KL, Weber JL, Unger MJ, Ledesma J, Yu N, Gassmann M, Lai C (1997). Neuregulin-2, a new ligand of ErbB3/ErbB4-receptor tyrosine kinases. Nature.

[6] Sundvall M, Iljin K, Kilpinen S, Sara H, Kallioniemi OP, Elenius K (2008). Role of ErbB4 in breast cancer. J Mammary Gland Biol Neoplasia.

[7] Carraway KL, Carraway CA, Carraway KL (1997). Roles of ErbB-3 and ErbB-4 in the physiology and pathology of the mammary gland. J Mammary Gland Biol Neoplasia.

[8] Naresh A, Long W, Vidal GA, Wimley WC, Marrero L, Sartor CI, Tovey S, Cooke TG, Bartlett JM, Jones FE (2006). The ERBB4/HER4 intracellular domain 4ICD is a BH3-only protein promoting apoptosis of breast cancer cells. Cancer Res.

[9] Sartor CI, Zhou H, Kozlowska E, Guttridge K, Kawata E, Caskey L, Harrelson J, Hynes N, Ethier S, Calvo B, Earp HS (2001). Her4 mediates ligand-dependent antiproliferative and differentiation responses in human breast cancer cells. Mol Cell Biol.

[10] Kainulainen V, Sundvall M, Maatta JA, Santiestevan E, Klagsbrun M, Elenius K (2000). A natural ErbB4 isoform that does not activate phosphoinositide 3-kinase mediates proliferation but not survival or chemotaxis. J Biol Chem.

[11] Suo Z, Risberg B, Kalsson MG, Willman K, Tierens A, Skovlund E, Nesland JM (2002). EGFR family expression in breast carcinomas. c-erbB-2 and c-erbB-4 receptors have different effects on survival. J Pathol.

[12] Machleidt A, Buchholz S, Diermeier-Daucher S, Zeman F, Ortmann O, Brockhoff G (2013). The prognostic value of Her4 receptor isoform expression in triple-negative and Her2 positive breast cancer patients. BMC Cancer.

[13] Barnes NL, Khavari S, Boland GP, Cramer A, Knox WF, Bundred NJ (2005). Absence of HER4 expression predicts recurrence of ductal carcinoma in situ of the breast. Clin Cancer Res.

[14] Koutras AK, Kalogeras KT, Dimopoulos MA, Wirtz RM, Dafni U, Briasoulis E, Pectasides D, Gogas H, Christodoulou C, Aravantinos G, Zografos G, Timotheadou E, Papakostas P, Linardou H, Razis E, Economopoulos T (2008). Evaluation of the prognostic and predictive value of HER family mRNA expression in high-risk early breast cancer: a Hellenic Cooperative Oncology Group (HeCOG) study. Br J Cancer.

[15] Witton CJ, Reeves JR, Going JJ, Cooke TG, Bartlett JM (2003). Expression of the HER1-4 family of receptor tyrosine kinases in breast cancer. J Pathol.

[16] Cohen BD, Kiener PA, Green JM, Foy L, Fell HP, Zhang K (1996). The relationship between human epidermal growth-like factor receptor expression and cellular transformation in NIH3T3 cells. J Biol Chem.

[17] Junttila TT, Sundvall M, Lundin M, Lundin J, Tanner M, Harkonen P, Joensuu H, Isola J, Elenius K (2005). Cleavable ErbB4 isoform in estrogen receptor-regulated growth of breast cancer cells. Cancer Res.

[18] Tang CK, Concepcion XZ, Milan M, Gong X, Montgomery E, Lippman ME (1999). Ribozyme-mediated down-regulation of ErbB-4 in estrogen receptor-positive breast cancer cells inhibits proliferation both in vitro and in vivo. Cancer Res.

[19] Bieche I, Onody P, Tozlu S, Driouch K, Vidaud M, Lidereau R (2003). Prognostic value of ERBB family mRNA expression in breast carcinomas. Int J Cancer.

[20] Portier BP, Minca EC, Wang Z, Lanigan C, Gruver AM, Downs-Kelly E, Budd GT, Tubbs RR (2013). HER4 expression status correlates with improved outcome in both neoadjuvant and adjuvant Trastuzumab treated invasive breast carcinoma. Oncotarget.

[21] Sassen A, Diermeier-Daucher S, Sieben M, Ortmann O, Hofstaedter F, Schwarz S, Brockhoff G (2009). Presence of HER4 associates with increased sensitivity to Herceptin in patients with metastatic breast cancer. Breast Cancer Res.

[22] Kong A, Calleja V, Leboucher P, Harris A, Parker PJ, Larijani B (2008). HER2 oncogenic function escapes EGFR tyrosine kinase inhibitors via activation of alternative HER receptors in breast cancer cells. PLoS One.

[23] Gijsen M, King P, Perera T, Parker PJ, Harris AL, Larijani B, Kong A (2010). HER2 phosphorylation is maintained by a PKB negative feedback loop in response to anti-HER2 herceptin in breast cancer. PLoS Biol.

[24] Kramer-Marek G, Gijsen M, Kiesewetter DO, Bennett R, Roxanis I, Zielinski R, Kong A, Capala J (2012). Potential of PET to Predict the Response to Trastuzumab Treatment in an ErbB2-Positive Human Xenograft Tumor Model. J Nucl Med.

[25] Rio C, Buxbaum JD, Peschon JJ, Corfas G (2000). Tumor necrosis factor-alpha-converting enzyme is required for cleavage of erbB4/HER4. J Biol Chem.

[26] Ni CY, Murphy MP, Golde TE, Carpenter G (2001). gamma-Secretase cleavage and nuclear localization of ErbB-4 receptor tyrosine kinase. Science.

[27] Wissner A, Mansour TS (2008). The development of HKI-272 and related compounds for the treatment of cancer. Arch Pharm (Weinheim).

[28] Canonici A, Gijsen M, Mullooly M, Bennett R, Bouguern N, Pedersen K, O'Brien NA, Roxanis I, Li JL, Bridge E, Finn R, Siamon D, McGowan P, Duffy MJ, O'Donovan N, Crown J (2013). Neratinib overcomes trastuzumab resistance in HER2 amplified breast cancer. Oncotarget.

[29] Maatta JA, Sundvall M, Junttila TT, Peri L, Laine VJ, Isola J, Egeblad M, Elenius K (2006). Proteolytic cleavage and phosphorylation of a tumor-associated ErbB4 isoform promote ligand-independent survival and cancer cell growth. Mol Biol Cell.

[30] Muraoka-Cook RS, Sandahl MA, Strunk KE, Miraglia LC, Husted C, Hunter DM, Elenius K, Chodosh LA, Earp HS (2009). ErbB4 splice variants Cyt1 and Cyt2 differ by 16 amino acids and exert opposing effects on the mammary epithelium in vivo. Mol Cell Biol.

[31] Muraoka-Cook RS, Sandahl M, Husted C, Hunter D, Miraglia L, Feng SM, Elenius K, Earp HS (2006). The intracellular domain of ErbB4 induces differentiation of mammary epithelial cells. Mol Biol Cell.

[32] Linggi B, Carpenter G (2006). ErbB-4 s80 intracellular domain abrogates ETO2-dependent transcriptional repression. J Biol Chem.

[33] Sundvall M, Peri L, Maatta JA, Tvorogov D, Paatero I, Savisalo M, Silvennoinen O, Yarden Y, Elenius K (2007). Differential nuclear localization and kinase activity of alternative ErbB4 intracellular domains. Oncogene.

[34] Ward TM, Iorns E, Liu X, Hoe N, Kim P, Singh S, Dean S, Jegg AM, Gallas M, Rodriguez C, Lippman M, Landgraf R, Pegram MD (2013). Truncated p110 ERBB2 induces mammary epithelial cell migration, invasion and orthotopic xenograft formation, and is associated with loss of phosphorylated STAT5. Oncogene.

[35] Recupero D, Daniele L, Marchio C, Molinaro L, Castellano I, Cassoni P, Righi A, Montemurro F, Sismondi P, Biglia N, Viale G, Risio M, Sapino A (2013). Spontaneous and pronase-induced HER2 truncation increases the trastuzumab binding capacity of breast cancer tissues and cell lines. J Pathol.

[36] Adilakshmi T, Ness-Myers J, Madrid-Aliste C, Fiser A, Tapinos N (2011). A nuclear variant of ErbB3 receptor tyrosine kinase regulates ezrin distribution and Schwann cell myelination. J Neurosci.

[37] Sundvall M, Veikkolainen V, Kurppa K, Salah Z, Tvorogov D, van Zoelen EJ, Aqeilan R, Elenius K (2010). Cell death or survival promoted by alternative isoforms of ErbB4. Mol Biol Cell.

[38] Williams CC, Allison JG, Vidal GA, Burow ME, Beckman BS, Marrero L, Jones FE (2004). The ERBB4/HER4 receptor tyrosine kinase regulates gene expression by functioning as a STAT5A nuclear chaperone. J Cell Biol.

[39] Elenius K, Choi CJ, Paul S, Santiestevan E, Nishi E, Klagsbrun M (1999). Characterization of a naturally occurring ErbB4 isoform that does not bind or activate phosphatidyl inositol 3-kinase. Oncogene.

[40] Aqeilan RI, Donati V, Gaudio E, Nicoloso MS, Sundvall M, Korhonen A, Lundin J, Isola J, Sudol M, Joensuu H, Croce CM, Elenius K (2007). Association of Wwox with ErbB4 in breast cancer. Cancer Res.

[41] Zhu Y, Sullivan LL, Nair SS, Williams CC, Pandey AK, Marrero L, Vadlamudi RK, Jones FE (2006). Coregulation of estrogen receptor by ERBB4/HER4 establishes a growth-promoting autocrine signal in breast tumor cells. Cancer Res.

[42] Feng SM, Sartor CI, Hunter D, Zhou H, Yang X, Caskey LS, Dy R, Muraoka-Cook RS, Earp HS (2007). The HER4 cytoplasmic domain, but not its C terminus, inhibits mammary cell proliferation. Mol Endocrinol.

[43] Olsauskas-Kuprys R, Zlobin A, Osipo C (2013). Gamma secretase inhibitors of Notch signaling. Onco Targets Ther.

[44] Schott A.F., Landis M.D., Dontu G., Griffith K.A., Layman R.M., Krop I., Paskett L.A., Wong H., Dobrolecki L.E., Lewis M.T. (2013). Preclinical and clinical studies of gamma secretase inhibitors with docetaxel on human breast tumors. Clin Cancer Res.

[45] Rabindran SK, Discafani CM, Rosfjord EC, Baxter M, Floyd MB, Golas J, Hallett WA, Johnson BD, Nilakantan R, Overbeek E, Reich MF, Shen R, Shi X, Tsou HR, Wang YF, Wissner A (2004). Antitumor activity of HKI-272, an orally active, irreversible inhibitor of the HER-2 tyrosine kinase. Cancer Res.

[46] Burstein HJ, Sun Y, Dirix LY, Jiang Z, Paridaens R, Tan AR, Awada A, Ranade A, Jiao S, Schwartz G, Abbas R, Powell C, Turnbull K, Vermette J, Zacharchuk C, Badwe R (2010). Neratinib, an irreversible ErbB receptor tyrosine kinase inhibitor, in patients with advanced ErbB2-positive breast cancer. J Clin Oncol.

[47] Thor AD, Edgerton SM, Jones FE (2009). Subcellular localization of the HER4 intracellular domain, 4ICD, identifies distinct prognostic outcomes for breast cancer patients. Am J Pathol.

[48] Tovey SM, Witton CJ, Bartlett JM, Stanton PD, Reeves JR, Cooke TG (2004). Outcome and human epidermal growth factor receptor (HER) 1-4 status in invasive breast carcinomas with proliferation indices evaluated by bromodeoxyuridine labelling. Breast Cancer Res.

[49] Sassen A, Rochon J, Wild P, Hartmann A, Hofstaedter F, Schwarz S, Brockhoff G (2008). Cytogenetic analysis of HER1/EGFR, HER2, HER3 and HER4 in 278 breast cancer patients. Breast Cancer Res.

[50] Veikkolainen V, Vaparanta K, Halkilahti K, Iljin K, Sundvall M, Elenius K (2011). Function of ERBB4 is determined by alternative splicing. Cell Cycle.

[51] Hollmen M, Liu P, Kurppa K, Wildiers H, Reinvall I, Vandorpe T, Smeets A, Deraedt K, Vahlberg T, Joensuu H, Leahy DJ, Schoffski P, Elenius K (2012). Proteolytic processing of ErbB4 in breast cancer. PLoS One.

[52] Sundvall M, Korhonen A, Paatero I, Gaudio E, Melino G, Croce CM, Aqeilan RI, Elenius K (2008). Isoform-specific monoubiquitination, endocytosis, and degradation of alternatively spliced ErbB4 isoforms. Proc Natl Acad Sci U S A.

[53] Mill CP, Zordan MD, Rothenberg SM, Settleman J, Leary JF, Riese DJ (2012). ErbB2 Is Necessary for ErbB4 Ligands to Stimulate Oncogenic Activities in Models of Human Breast Cancer. Genes Cancer.

[54] Waterhouse B, Gijsen M, Barber P, Tullis I, Vojnovic B, Kong A (2011). Assessment of EGFR/HER2 dimerization by FRET-FLIM utilizing Alexa-conjugated secondary antibodies in relation to targeted therapies in cancers. Oncotarget.

