# Supplementary material for: Nuclear HER4 mediates acquired resistance to trastuzumab and is associated with poor outcome in HER2 positive breast cancer
Source: Oncotarget. 2014 Apr 17;5(15):5934–49. doi: 10.18632/oncotarget.1904 (PMC4171603; doi:10.18632/oncotarget.1904)
Supplement: Supplementary file 1 [file oncotarget-05-5934-s001.pdf]

Nuclear HER4 mediates acquired resistance to trastuzumab and is associated with poor outcome in HER2 positive breast cancer

Supplementary Material

Supplementary Table 1: Correlations of cytoplasmic and nuclear HER4 expression with the patient characteristics and tumour histology in a cohort of HER2 positive breast cancer patients.

Characteristics		Cytoplasmic HER4			Nuclear HER4		
		LOW (<6)	HIGH (≥6)		NEG (0)	POS (≥1)	
Tumour grade 1		11	10				
	2	24	28		12	9	
	3	35	38		35	17	
				p=0.796	47	26	p=0.430
Tumour size							
	<2 cm	12	12		16	8	
	≥2 cm	22	26		31	17	
		34	38		47	25	p=1.000
				p=0.805			
Age							
	<55 years	10	16		19	6	
	≥55 years	25	22		27	20	
		0	38	p=0.328	46	26	p=0.132
Menopausal							
	Pre	6	9		12	3	
	Post	29	29		35	23	
		35	38	p=0.569	47	26	p=0.228
Nodes							
	Neg	15	21		23	13	
	Pos	16	17		23	10	
		31	38	0=0.632	46	23	p=0.798
Estrogen receptor							
	Neg	6	14		14	5	
	Pos	27	24		31	20	
		33	38	p=0.113	45	25	p=0.405

Supplementary Table 2: Multivariate analysis for cytoplasmic and nuclear HER4 expression in a cohort of HER2 positive breast cancer patients.

**Variables**	**OS (n=73)**		**RFS (n=73)**	
	HR (95% CI)	*P*-value	HR (95% CI)	*P*-value
Cytoplasmic HER4	0.240 (0.55-1.054)	0.059	0.313 (0.96-1.022)	0.054
Nodes	4.398 (0.512-37.794)	0.177	2.713 (0.715-10.298)	0.143
ER	1.042 (0.245-4.421)	0.956	0.427 (0.133-1.372)	0.153
Nuclear HER4	14.281 (2.722-74.928)	0.002	5.573 (1.1474-21.077)	0.011
Nodes	6.613 (0.752-58.132)	0.089	4.194 (1.117-15.740)	0.034
ER	.980 (0.220-4.365)	0.979	0.519 (0.151-1.788)	0.299

P-values and hazard ratio (HR) were calculated from Cox proportional hazards regression models adjusted for ER and Nodal status.

Supplementary Table 3: Baseline patient characteristics with clinical and tumour parameters of HER2 positive patients underwent a window study of trastuzumab monotherapy and neoadjuvant therapy.

Characteristics	Value
Median age (range)	59 (39-79)
Median clinical tumour size (range)	40 (15-80)
Tumour grade	
1, 2	5 (29%)
3	12 (71%)
Menopausal status	
Pre	5 (29%)
Post	12 (71%)
ER	
Negative	8 (47%)
Positive	9 (53%)
PR	
Negative	5 (29%)
Positive	12 (71%)

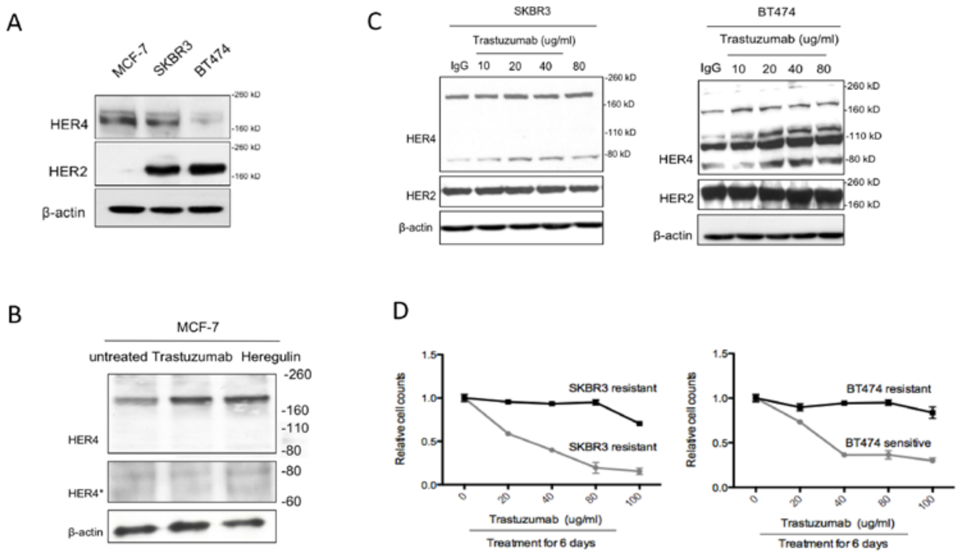

Supplementary Figure 1: The effect of trastuzumab and heregulin on HER4 expression in HER2 positive and negative breast cancer cells. (A) The expression of HER4 and HER2 in MCF-7, SKBR3 and BT474 cells was analysed using western blot analysis. β-actin was used as the loading control. (B) MCF-7 cells were stimulated with 100ng/ml heregulin or treated with 40μg/ml trastuzumab for one-hour duration before western blot analysis for the indicated proteins. (*) indicates that the membrane was exposed in a longer time to obtain the bands at 80 kDa. β-actin was used as the loading control. (C) The figures shows HER4 expression by western blot in SKBR3 and BT474 cells treated with increasing doses of trastuzumab as indicated. IgG was used as a treatment control. (D) The cell count experiments were done in sensitive and resistant SKBR3 and BT474 cells after 6 days' treatment with increasing doses of trastuzumab as indicated.

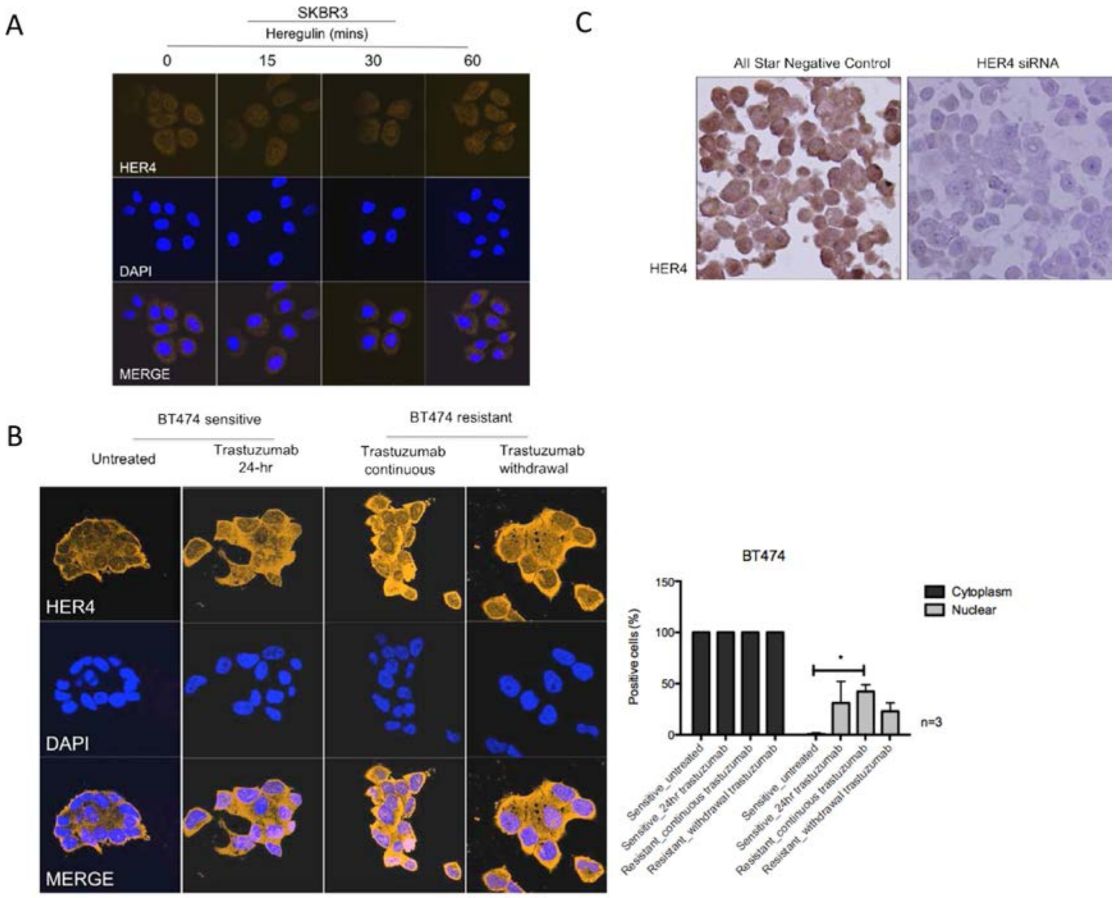

Supplementary Figure 2: The effect of heregulin and trastuzumab on HER4 localisation in SKBR3 and BT474. (A) Representative confocal images of HER4 expression from SKBR3 cells stimulated with 100ng/ml heregulin for the indicated times. DAPI staining was used to indicate nuclear localisation. (B) Representative confocal images from sensitive and acquired resistant BT474 cells treated with 40µg/ml trastuzumab are shown. The right panel shows the percentage of positive cells stained with HER4-Alexa Fluor 546 at cytoplasmic and nuclear localisation. Means ± SD from three independent experiments are shown in the graph. 1-way ANOVA test with Bonferroni's multiple comparisons was applied to determined significant differences of HER4 protein between groups (*p<0.05). (C) The IHC staining using anti-HER4 antibody (Neomarkers) in paraffin-embedded cell pellets made from either the scramble or HER4 siRNA-transfected SKBR3 cells.

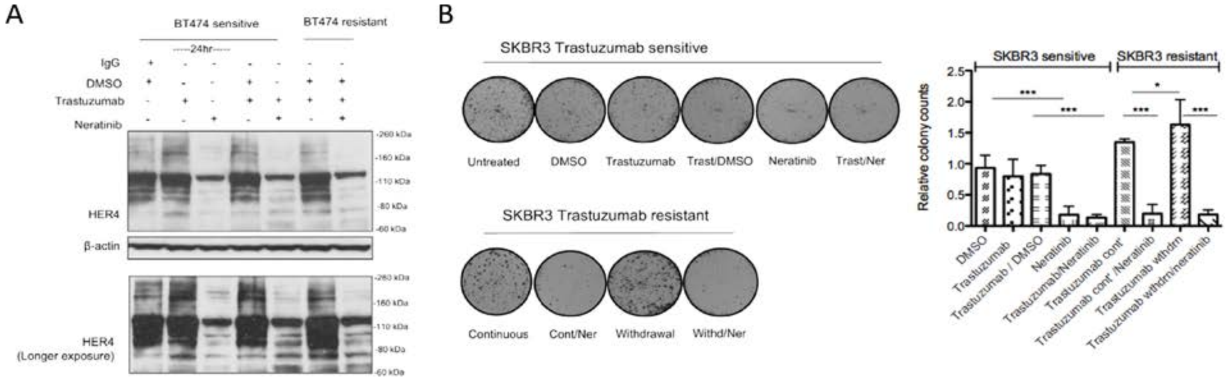

Supplementary Figure 3: Neratinib enhances trastuzumab sensitivity and reverses its resistance in HER2 positive breast cancer cells. (A) Representative western blots of HER4 of sensitive and resistant BT474 cells treated with 100 nM neratinib and/or 40 μg/ml trastuuzmab are shown. (B) SKBR3 cells were treated with 100 nM neratinib or/and 40 μg/ml trastuzumab treatment for 72 hours being trypsinised and replated in duplicate for colony-formation assay for 7 days. Representative photographs of the different conditions are shown on the middle panels and the relative ratio of colony counts (means ± SD) is shown on the right panel (*p<0.05, ***p<0.001).

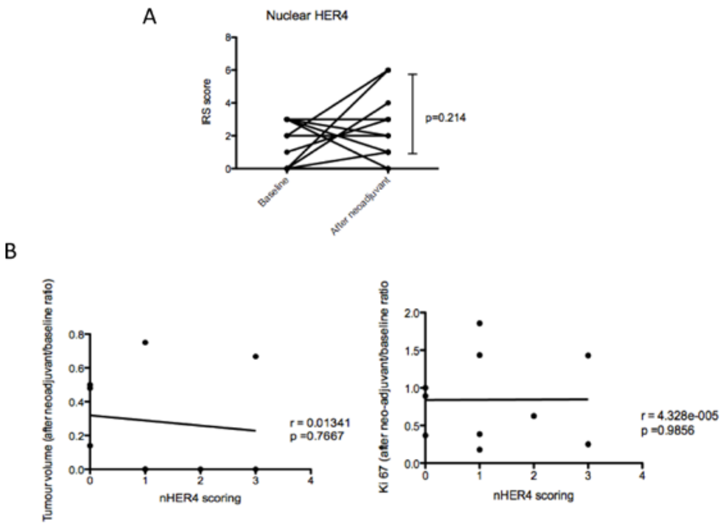

Supplementary Figure 4: HER4 expression after neo-adjuvant chemotherapy and trastuzumab treatment in HER2 positive breast cancer patients. (A) Nuclear HER4 expression in tumour samples from patients who received neoadjuvant chemotherapy with trastuzumab was compared to that obtained from the baseline. Analysis on HER4 IRS scores was done in paired tissue samples from 14 patients using paired t-test. (B) Nuclear HER4 expression of tumours from definitive surgical samples (after HER2 positive breast cancer patients underwent neoadjuvant chemotherapy with trastuzumab) was correlated with the ratios of Ki67 staining and tumour volume (between definitive surgery and baseline) by linear regression.
